# Estimating the effective reproduction number of HIV infections in Japan from 1985 to 2025: a Bayesian extended back calculation

**DOI:** 10.3389/fpubh.2026.1944492

**Published:** 2026-10-27

**Authors:** Seiko Fujiwara, Hiroshi Nishiura

**Affiliations:** 1 Department of Epidemic Intelligence and Health Emergency Response, Center for Health Security, Graduate School of Medicine, Kyoto University, Kyoto, Japan; 2 School of Public Health, Graduate School of Medicine, Kyoto University, Kyoto, Japan; 3 Department of Epidemiology and Biostatistics, Schulich School of Medicine & Dentistry, Western University, London, ON, Canada

**Keywords:** AIDS, control, evaluation, Japan, mathematical model, men who have sex with men, transmissibility

## Abstract

**Background:**

HIV transmission among men having sex with men remains a concerning issue in Japan. Therefore, the purpose of the present study was to estimate the effective reproduction number *R*(*t*) of HIV by coupling a renewal process model with an existing extended back-calculation model in a Bayesian framework.

**Methods:**

We analyzed the annual number of HIV diagnoses and AIDS cases among Japanese males from 1985 through 2025. Using a Bayesian back-calculation with penalized *B*-splines for the incidence and the diagnosis rate of HIV with a renewal equation, we estimated *R*(*t*) for Japan and different geographic regions within the country.

**Results:**

From 2008 to 2010, we observed a consistent median value of *R*(*t*) < 1, with the most recent estimate of *R*(*t*) being the most uncertain. *R*(*t*) < 1 was apparent in major metropolitan areas, including Tokyo, Kanto–Koshinetsu, and Kinki, where even the upper bound of the 95% credible interval fell below 1 in the early-to-mid 2010s. The annual HIV diagnosis rate declined from shortly before the start of the COVID-19 pandemic, and the estimated proportion of HIV-infected individuals has increased only slowly in recent years, with the proportion of diagnosed individuals not reaching 95% or even 90% of the true total.

**Conclusion:**

Our general *R*(*t*) value indicates that the control of HIV/AIDS in Japan has been successful over the long term, including during the COVID-19 pandemic. However, the uncertainty of recent *R*(*t*) values suggests the need for regular updates of the estimate; reforming the testing and treatment strategies in Japan is vital to accomplishing this goal.

## Introduction

Acquired immunodeficiency syndrome (AIDS) is a retroviral infection caused by the human immunodeficiency virus (HIV) (1) following a median incubation period of approximately 10 years, with many infections going undiagnosed for a number of years (2, 3). Viral suppression by antiretroviral therapy can prevent secondary transmission (leading to the idea that an undetectable viral load is equal to untransmissible (i.e., U=U) and the concept of ‘treatment as prevention’ being a mainstay of disease control) (4, 5). As a slogan, the 95–95–95 continuum of care has been proposed by the Joint United Nations Program on HIV/AIDS (UNAIDS) as the target goal by 2030 as a way to eventually eliminate HIV infection (6, 7). In 2024, a global estimate of 40.8 million people were thought to be living with HIV, with 5.3 million unaware of their infection status (6).

In Japan, despite advances in diagnosis and treatment, secondary transmission has persisted, presumably from infected individuals unaware of their infection (3, 8). The proportion of individuals in Japan with HIV who have been diagnosed was estimated to be 89.3% at the end of 2022 (8, 9), which fell short of the 95% UNAIDS target. Moreover, the number of diagnosed AIDS cases increased for a second consecutive year in 2024, while the proportion of AIDS cases among newly notified infections reached its highest level in two decades (10). However, whether HIV transmission is under control cannot be directly inferred by looking only at counts of diagnosed HIV infections and AIDS cases. Instead, it requires the effective reproduction number *R*(*t*), defined as the average number of secondary transmissions generated by a single infected individual at calendar time *t* (11, 12). In Japan, routine monitoring of *R*(*t*) has yet to be achieved, including its use in evaluating regional epidemiological situations.

In Japan, newly diagnosed HIV infections and AIDS cases have been under regional surveillance since 1985 (10). Therefore, extended back-calculations can help reconstruct the actual incidence of new HIV infections—including individuals yet to be diagnosed—and the diagnosis rate over time from the notification data (3, 13, 14). Theoretically, *R*(*t*) can then be derived from a renewal equation by analyzing the reconstructed incidence (15–17) while Bayesian methods can help compute the uncertainties surrounding the estimates.

Therefore, the purpose of the present study was to estimate the effective reproduction number *R*(*t*) by coupling a renewal process model with an existing extended, back-calculation model. A Bayesian estimation method was then used, and *R*(*t*) was estimated at the national and regional levels.

## Materials and methods

According to the Infectious Disease Control Law of Japan, HIV and AIDS are designated as category V notifiable diseases; thus, all physicians who diagnose an infected individual must notify the government (18). We analyzed the surveillance data published by the Committee of AIDS Trends at the Ministry of Health, Labour and Welfare (10). The dataset covered the years from 1985 to the end of 2025, and the data were broken down by sex, age group, and nationality. An individual was diagnosed as infected with HIV when a positive antibody screening test using an enzyme-linked immunosorbent assay was confirmed with an HIV-1/HIV-2 differentiation assay and/or an HIV-1 RNA nucleic acid assay test. AIDS is diagnosed when an HIV-infected person had one of 23 designated indicator diseases (19). By the end of 2025, a total of 25,815 HIV diagnoses and 11,446 AIDS cases had been reported (10).

We analyzed the yearly count of HIV diagnoses and newly diagnosed AIDS cases among Japanese nationals. In Japan, infected individuals are primarily men (e.g., they accounted for more than 96% of cases for both HIV and AIDS in 2024 and more than 97% in 2023), with the majority of cases likely caused by transmission in populations of men who have sex with men (MSM) (10). Therefore, in the present study, we focused on the transmission dynamics in Japanese men and analyzed nationwide data collected through 2025. For regional analyses, prefectures were aggregated into eight blocks. Regional datasets were available up to the end of 2024.

The following four steps were taken to estimate *R*(*t*): (i) we designed a model to estimate the incidence of new HIV infections *λ*(*t*) and the diagnosis rate *α*(*t*), smoothing them using spline functions; (ii) using the extended back-calculation method (3, 14), we estimated parameters via the Bayesian method, fitting the model to two observed series data (i.e., HIV diagnoses and AIDS cases) and allowing for Poisson-distributed variation in every year; (iii) assuming that the generation-time distribution was known, we derived the estimate of the effective reproduction number *R*(*t*) for each posterior draw through a renewal equation (15–17); and (iv) we extended the nationwide approach to a regional hierarchical model (with and without spatial dependence) and identified the best model via model comparison.

For the mathematical modelling of steps (i)–(iii), our exercise took two distinct approaches. First, an extended back-calculation model was used to reconstruct the incidence of HIV infection, accounting for the incubation period and estimating the yearly rate of diagnosis. This step did not involve an explicit estimation of the transmission process, and *λ*(*t*) and *α*(*t*) were estimated as unknown quantities. Second, the reconstructed incidence was modelled using the renewal equation to derive the estimate of *R*(*t*). Appropriately accounting for the generation time that is determined by the relative infectiousness *β*(*s*), the process of secondary transmission was captured. Supplementary Table S2 summarizes all variables and parameters that we handled in the present study.

Here, we briefly describe the key technical points in each step; mathematical details are provided in the supporting material. First, *λ*(*t*) and *α*(*t*) were described either by step-function or smooth functions of calendar time using penalized cubic *B*-splines (20, 21); the basis expansion and the second-order random-walk smoothing priors are described in Supplementary material S4.

Second, an individual infected in year *t* − *s* (still undiagnosed without yet having developed AIDS) enters year *t* at infection age *s*. Using the discretized McKendrick equation whose full derivation can be found elsewhere (3) and is reproduced in Supplementary material S2, the expected numbers of new HIV and AIDS diagnoses in year *t* are described as follows Equations 1, 2:


$$E[{\mathrm{HIV}}_{t}]=\alpha_{t}\sum_{s=1}^{t-1}\lambda_{t-s}S_{\rho }(s)\prod_{x=t-s+1}^{t-1}(1-\alpha_{x}),$$ (1)

$$E[{\text{AIDS}}_{t}]=\sum_{s=1}^{t-1}\lambda_{t-s}f_{\rho }(s)\prod_{x=t-s+1}^{t-1}(1-\alpha_{x}).$$ (2)

where *λ_t − s_* is the yearly incidence of new HIV infections in year *t* − *s*, *α_t_* is the yearly diagnosis probability, and $\prod_{x=t-s+1}^{t-1}(1-\alpha_{x})$ is the probability of having remained undiagnosed by year *t*−1. The yearly variations in observed HIV and AIDS diagnosis counts were assumed to be sufficiently described by a Poisson distribution. Fitting the model to two observed time series jointly helped identify *λ*(*t*) and *α*(*t*) (22). *S_ρ_*(*s*) represents AIDS-free survival, whereas *f_ρ_*(*s*) = *S_ρ_*(*s* − 1) − *S_ρ_*(*s*) gives the yearly probability of developing AIDS while the time since HIV infection ages from *s*−1 to *s*. The hazard of developing AIDS was derived from the incubation period that was inferred from clinical studies and was modeled using a discrete Weibull distribution with a scale parameter *η* = 11.596 years and a shape parameter *k* = 2.516, corresponding to a median incubation period of 10.02 years (2, 23, 24). The parameter values are summarized in Supplementary Table S1.

The two observed series were assumed to be sufficiently described by Poisson distributions. The likelihood was constructed by multiplying the Poisson probabilities for both series across all years from 1985 through 2025. Full specifications of the likelihood and prior distributions are given in Supplementary materials S1, S4.

Third, we assumed that only undiagnosed individuals contributed to transmission. For an infector of infection age *s* (years since infection), the relative infectiousness *β*(s) followed a three-phase step profile over infection age (acute, chronic, and late) (25, 26). The transmission weight was the product of *β*(s), AIDS-free survival *S_ρ_*(s), and the undiagnosed survival *S_α_*(*t,s*), giving the normalized weights *w*_s,t_∝ *β*(*s*)*S_ρ_*(*s*)*S_α_*(*t*,*s*) where *w*_s,t_ is the probability density of the generation time in year *t*. We specified *β*(*s*) and the infection-age boundaries adhering to those inferred by Abu-Raddad and Longini (25) and Hollingsworth et al. (26); all values are summarized in Supplementary Table S1. As we fixed *β*(*s*), we evaluated the sensitivity of our estimate to the generation-time. Using *w*_s,t_, we computed *R*(*t*) from each posterior draw of *λ* and *α*, summarized by the posterior median and 95% credible interval (95% CrI), using the following equation (Equation 3):


$$R_{t}=\frac{\lambda_{t}}{\sum_{s=1}^{s_{\max}}w_{s,t}\lambda_{t-s}}.$$ (3)

Given that up to *s*_max_ years (set as 10 years in the default due to greatly diminished transmissibility in and after *s* = 11 years) of prior history were required, *R*(*t*) was identifiable from approximately 1995 onward. As a complementary indicator of recent trends, we also computed the incidence-to-prevalence ratio, the reconstructed number of new HIV infections divided by the undiagnosed prevalence [i.e., *λ*(*t*)/*X*(*t*) for each posterior draw where *X*(*t*) is the undiagnosed prevalence of HIV infected individuals who have not developed AIDS yet] and summarized it using the posterior median and 95% CrI, which corresponded to part of the actual reproduction number (11, 27).

Fourth, the regional estimate of *R*(*t*) was modeled as regional deviations around the national mean. We compared three possible models to describe spatial dependence, nonspatial (independent), geographic adjacent, and MSM-venue adjacent, and adopted the best-performing, parsimonious nonspatial model (28, 29). The specifications and comparisons are detailed in the Supplementary material S7. To examine terminal, right-censoring effects in the regional estimates (as was done for the national series), we refitted the regional hierarchical model to data truncated at the years 2022, 2023, and 2024 (the regional series ended in 2024) and overlaid the resulting *R*(*t*). Not only employing the spline model for *λ*(*t*) and *α*(*t*), but we computed *R*(*t*) employing the piecewise constant model as described above.

Fifth, to examine whether the uncertainty of *R*(*t*) near the end of the series arose from the smoothing procedure, we performed the estimation of *λ* and *α*, using a piecewise constant model. The study period was partitioned into eleven calendar-time blocks, with four-year intervals from 1985 through 2016 and three-year intervals from 2017 onward. Models using different time steps of one, two, three and five years, as well as the specification described above, were compared using leave-one-out cross-validation, the widely applicable information criterion (WAIC), and the Bayesian information criterion (BIC) (Supplementary material S8). To compare the performance of two approaches (i.e., spline and piecewise constant models for *λ* and *α*), the validity (squared error), coverage probability and interval score were compared between the latest estimate in past years [e.g., estimated *R*(*t*) in 2021 obtained at the end of 2021] and the updated estimate as of the latest observation time at the end of 2025 [e.g., estimated *R*(*t*) in 2021 obtained at the end of 2025] (Supplementary material S8). The incidence-to-prevalence ratio was also derived using the piecewise constant model.

Posterior distributions were estimated with the Hamiltonian Monte Carlo (No-U-Turn Sampler) method using Stan software (30). For the sensitivity analysis, we assessed robustness of the assumed generation-time distribution by scaling its mean and standard deviation by factors of 0.70–1.30 (i.e., allowing plus/minus 30% variations) and re-estimating *R*(*t*) for each scenario. Sampler settings, convergence diagnostics (*R̂*, effective sample size) (31), and the baseline generation-time mean and standard deviation with the scaling grid are provided in the Supplementary material. All analyses were carried out using R 4.6.1 and Stan (cmdstanr) with model comparison via the loo package (32). The Supplementary material provides the surveillance data used for the national analysis together with the code required to reproduce all the results.

## Results

The back-calculation model was fitted to the data from 1985 through 2025. The 50 and 95% posterior predictive intervals successfully included the observed data points (Figures 1A,B). The estimated annual rate of HIV diagnoses *α*(*t*) rose from approximately 4% in 1985 to 16% in 2018 and slightly declined thereafter (Figure 1C), while the yearly incidence of new HIV infections *λ*(*t*) peaked in the early 2000s and monotonously decreased subsequently (Figure 1D). Convergence was satisfactory, i.e., for all 26 parameters *R̂* was below 1.01, and the bulk and tail effective sample sizes were well above the target of 400 (Supplementary Figure S1). Moreover, the four chains showed no separation, sticking, or trend (Supplementary Figure S2). In contrast, the posterior correlation between *λ* and *α* strengthened toward the most recent year of observation (falling below −0.7 at the end of the series, with an elongated joint posterior), reflecting the structurally weak identifiability of the parameters, which is a known and inherent limitation of the back-calculation method (Supplementary Figure S3).

**Figure 1:**
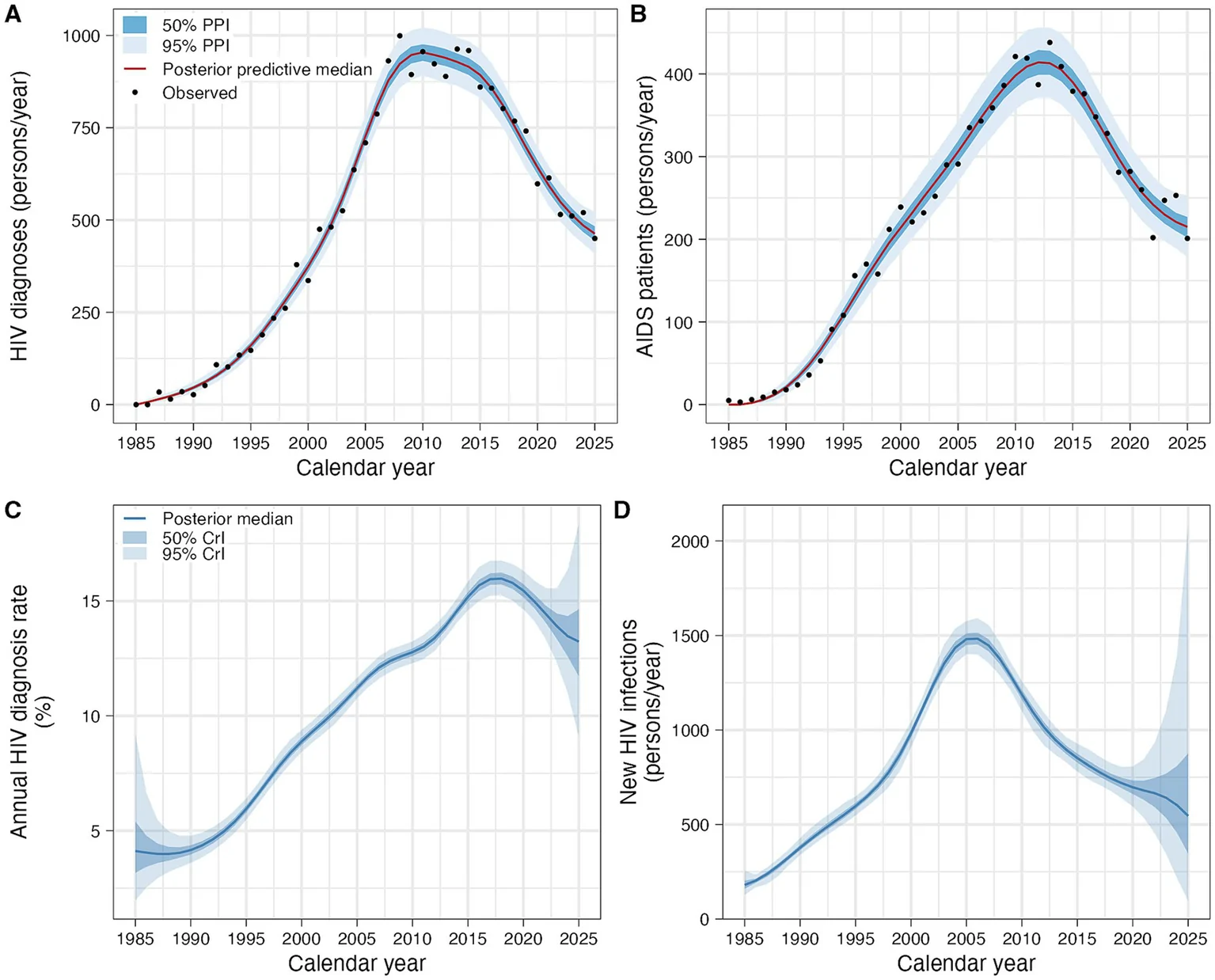
Annual number of new HIV diagnoses and AIDS cases in Japan, 1985–2025 along with model parameters. Posterior predictive fit to the annual number of reported **(A)** new HIV diagnoses and **(B)** AIDS cases. Points represent observed counts. The red line is the posterior predictive median, and the blue shaded bands are the 50 and 95% posterior predictive intervals. **(C)** Estimated annual HIV diagnosis rate, *α*(*t*), is shown as the posterior median with its 95% credible interval. **(D)** Estimated annual number of new HIV infections, *λ*(*t*). The posterior median with its 95% credible interval is shown.

Nationally, *R*(*t*) first fell below 1 in 2009, with a posterior median of 0.98 (95% CrI: 0.92, 1.02) (Figure 2A). Thereafter, the median consistently remained below 1 through 2025. Using the data up to 2025, *R*(*t*) rose slightly from 2015 and throughout the COVID-19 pandemic before turning downward from 2024, reaching 0.85 (95% CrI: 0.22, 2.01) in 2025. Truncating the series in 2024 yielded the latest estimate of *R*(*t*) above 1, but adding 2025 data returned the trend downward and yielded a median *R*(*t*) below 1. When the series was truncated in earlier years (Figure 2A), the latest *R*(*t*) always involved a wide credible interval, and the uncertainty bound crossed the value of 1. The latest estimate obtained with data through 2025 yielded a narrow uncertainty bound in earlier years and fell largely within the credible intervals that were estimated in the past. The rise or fall in the latest year reflected uncertainties rather than an actual resurgence or abrupt decline in *R*(*t*). The values of *λ*(*t*) in recent years was strongly correlated with *α*(*t*) (Supplementary Figure S3).

**Figure 2:**
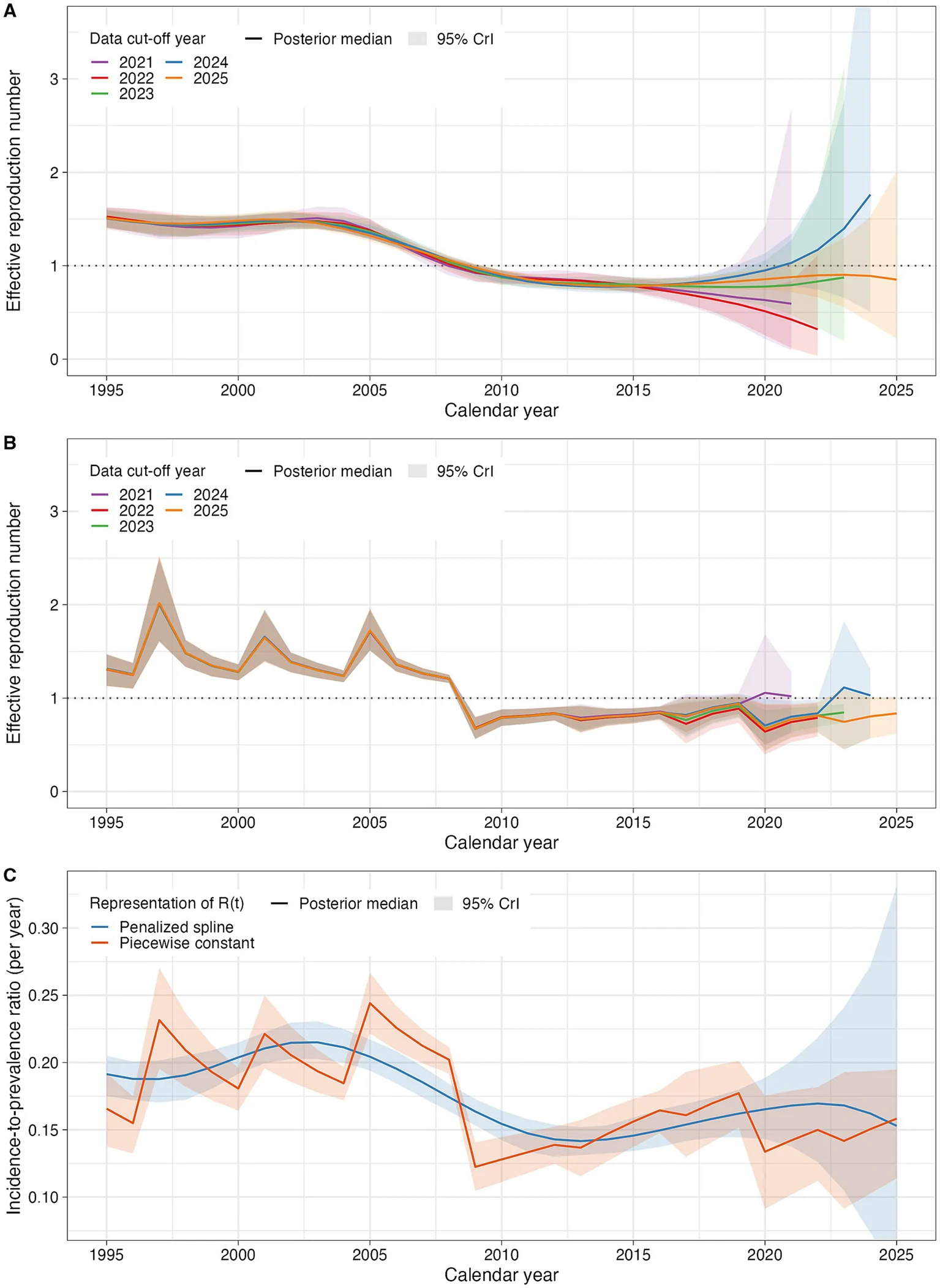
The effective reproduction number of HIV in Japan, 1985–2025 and the incidence-to-prevalence ratio. **(A)** The effective reproduction number *R*(*t*) estimated employing penalized-spline from the data series truncated at each calendar year from 2021 to 2025 and overlaid to show terminal sensitivity to right-censoring (color, data cut-off year; line, posterior median; shade, 95% credible interval); the dotted horizontal line indicates *R*(*t*) = 1. Five curves represent different data cut-off years. **(B)** *R*(*t*) estimated employing a piecewise constant model from the same datasets up to 2021–25. **(C)** The incidence-to-prevalence ratio; the reconstructed number of new HIV infections divided by the estimated undiagnosed prevalence, shown as the posterior median (line) with its 95% credible interval (shaded region). Both the spline model (blue) and piecewise constant model (orange) are shown.

Replacing the spline by piecewise constant model for *λ*(*t*) and *α*(*t*), the uncertainty bound of *R*(*t*) in the recent time was drastically reduced (Figure 2B). Using the observed data up to 2025, the width of 95% CrI was 0.39 using the piecewise constant model in contrast to 1.79 for spline model. However, the piecewise constant model still produced the latest posterior median *R*(*t*) estimates above the value of 1 in 2021 and 2024. Comparing the performance between spline and piecewise constant models, the piecewise constant model showed greater stability, with a root mean square error of 0.17 compared with 0.52 for the spline model (Supplementary Table S3; Supplementary Figure S4). The one- and two-year steps yielded extremely large interval scores (Supplementary Figure S4). Among the remaining specifications, the step function used in the main analysis performed the best across all three criteria (Supplementary Table S4). To further address uncertainty in recent estimates of *R*(*t*), we also calculated the incidence-to-prevalence ratio using both modelling approaches (Figure 2C). The posterior-median of the ratio from 2020 to 2025 ranged from 0.13 to 0.16 using the piecewise constant model and from 0.15 to 0.17 employing the spline, revealing a broadly consistent trend.

Among the three possible candidate models used to describe regional dependence, the nonspatial one was the best according to both elpd_LOO and WAIC, and the spatially structured share was near zero (i.e., there was no strong indication of spatial dependence) (Table 1). All of the regions followed a broadly common temporal pattern (Figure 3A), whereby they experienced a peak of *R*(*t*) > 1 from the late 1990s to the early 2000s, and every geographic region crossed over to a median *R*(*t*) < 1 by at least 2012. This decline, with narrow uncertainties, was identified in major metropolitan regions including Tokyo, Kinki, and Kanto-Koshinetsu. Hence, the median *R*(*t*), as well as the upper bound of *R*(*t*), took on a value below 1 for a period during the early to mid-2010s (i.e., all three regions from 2012 to 2014 and also spanning from 2009 to 2017 in Kinki). There was a notable rise in the latest estimates in Hokuriku and Chugoku–Shikoku, but they occurred in the latest time period, with a broad uncertainty bound crossing a value of 1 (Figure 3A). Employing the piecewise constant model for the HIV incidence and diagnosis rate, Figure 3B shows the alternative regional estimates of *R*(*t*). Uncertainties of *R*(*t*) in the latest timepoint decreased compared with spline model, including Hokuriku where recent estimates are not sharply pointing upward trend with piecewise constant model. However, supercritical timepoints of *R*(*t*) were seen in Chugoku-Shikoku and Kyushu-Okinawa with piecewise constant model, which were just covered by 95% credible intervals using spline, calling for a little longer time of observation to analyze more in detail.

**Table 1:** Comparison of the candidate models for the regional analyses.

Model	elpd_LOO	WAIC	BIC	Number of parameters	Spatially structured share
Nonspatial hierarchical	−2001.2	3987.6	5198.8	220	–
Spatial (scaled Besag-York-Mollié), geographic adjacency	−2001.9	3988.1	5824.7	317	0.02 (0–0.24)
Spatial (scaled Besag-York-Mollié), MSM-venue adjacency	−2002.7	3989.0	5820.9	317	0.11 (0–0.60)

Three candidate models were fitted to the same 640 observations (annual new HIV diagnoses and AIDS cases for each of the eight regions between 1985 and 2024): a nonspatial hierarchical model and two spatial (scaled Besag–York–Mollié) models that share information across regions and defined as adjacent either geographically or through shared MSM (men who have sex with men) venues. WAIC (Watanabe–Akaike Information Criterion) and BIC (Bayesian Information Criterion) are information criteria for which lower values indicate a better fit after model complexity is penalized. The spatially structured share is the estimated fraction of the between-region variation that is spatially structured and given as the posterior median with its 95% credible interval.

**Figure 3:**
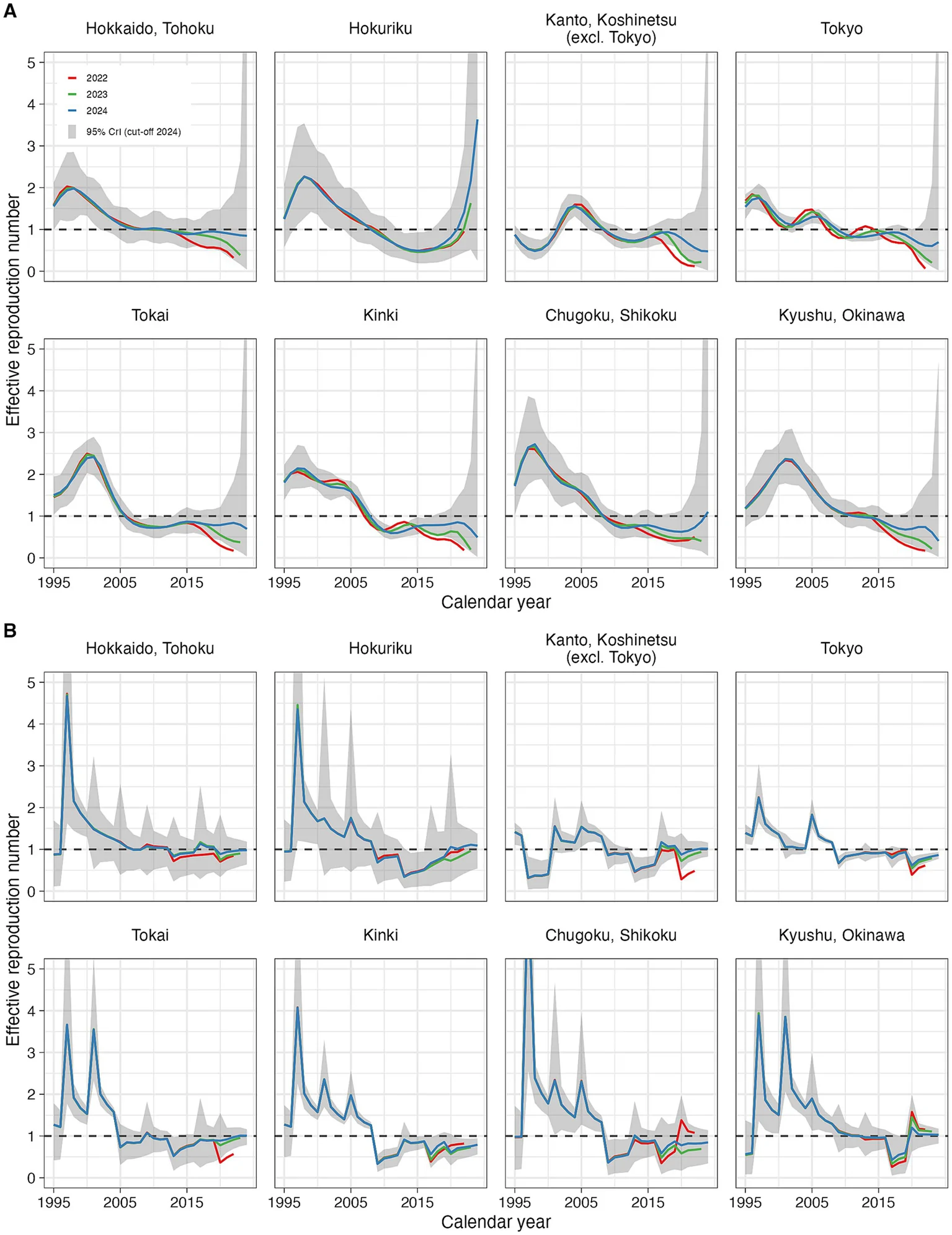
Regional estimates of the effective reproduction number *R*(*t*) in Japan, 1985–2024. **(A)** Spline model was employed for the HIV incidence and yearly diagnostic rate. To show terminal sensitivity to right-censoring, the regional posterior median is overlaid for three data cut-off years (color: 2022, 2023, 2024), and the shaded band shows the 95% credible interval for the 2024 cut-off. Regional deviations around the national mean were modeled with the nonspatial, hierarchical model (independent and identically distributed regional random effects); comparisons with the spatial (scaled Besag–York–Mollié) alternatives are summarized in Table 1. **(B)** Piecewise constant model for the incidence and diagnosis rate.

Although *R*(*t*) remained below 1, the diagnosis rate also declined (Figure 1C) and the proportion of HIV-infected individuals that had been diagnosed did not reach the 95% target in 2025 (Figure 4). As of 2025, the diagnosed proportion was 89.2% (95% CrI: 82.0, 93.0) even when all AIDS cases were included in the denominator (Figure 4A), and 85.0% (95% CrI: 75.8, 90.2) when restricted to HIV infections only (Figure 4B).

**Figure 4:**
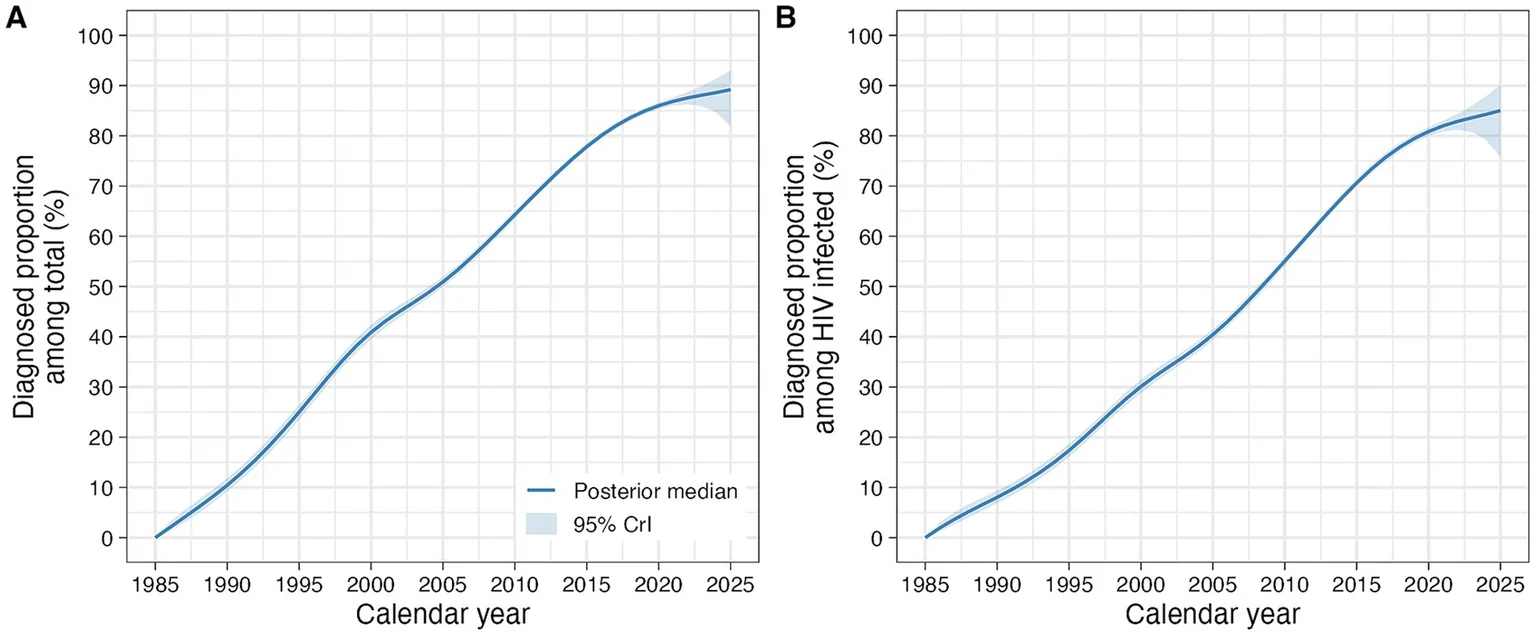
Estimated proportion of HIV-infected men who have been diagnosed. **(A)** Proportion of diagnosed individuals among all infected individuals, including those who have already developed AIDS. **(B)** Proportion of diagnosed individuals among HIV-infected individuals, excluding AIDS cases. Lines are posterior medians and shaded bands are 95% credible intervals.

When the mean and standard deviation of the generation time were varied, the *R*(*t*) trend was similar (Figure 5). While the variation was considerable when *R*(*t*) > 1 (e.g., before 2005), the effect became smaller in more recent years when *R*(*t*) = 1. Hence, *R*(*t*) was more sensitive to variations in the standard deviation than in the mean generation time.While the variation was considerable when R(t) > 1 (e.g., before 2005), the effect became smaller after *R*(*t*) fell below 1. *R*(*t*) was more sensitive to the mean than to the standard deviation before 2005, whereas the standard deviation had a relatively larger effect in the early to mid-2010s.

**Figure 5:**
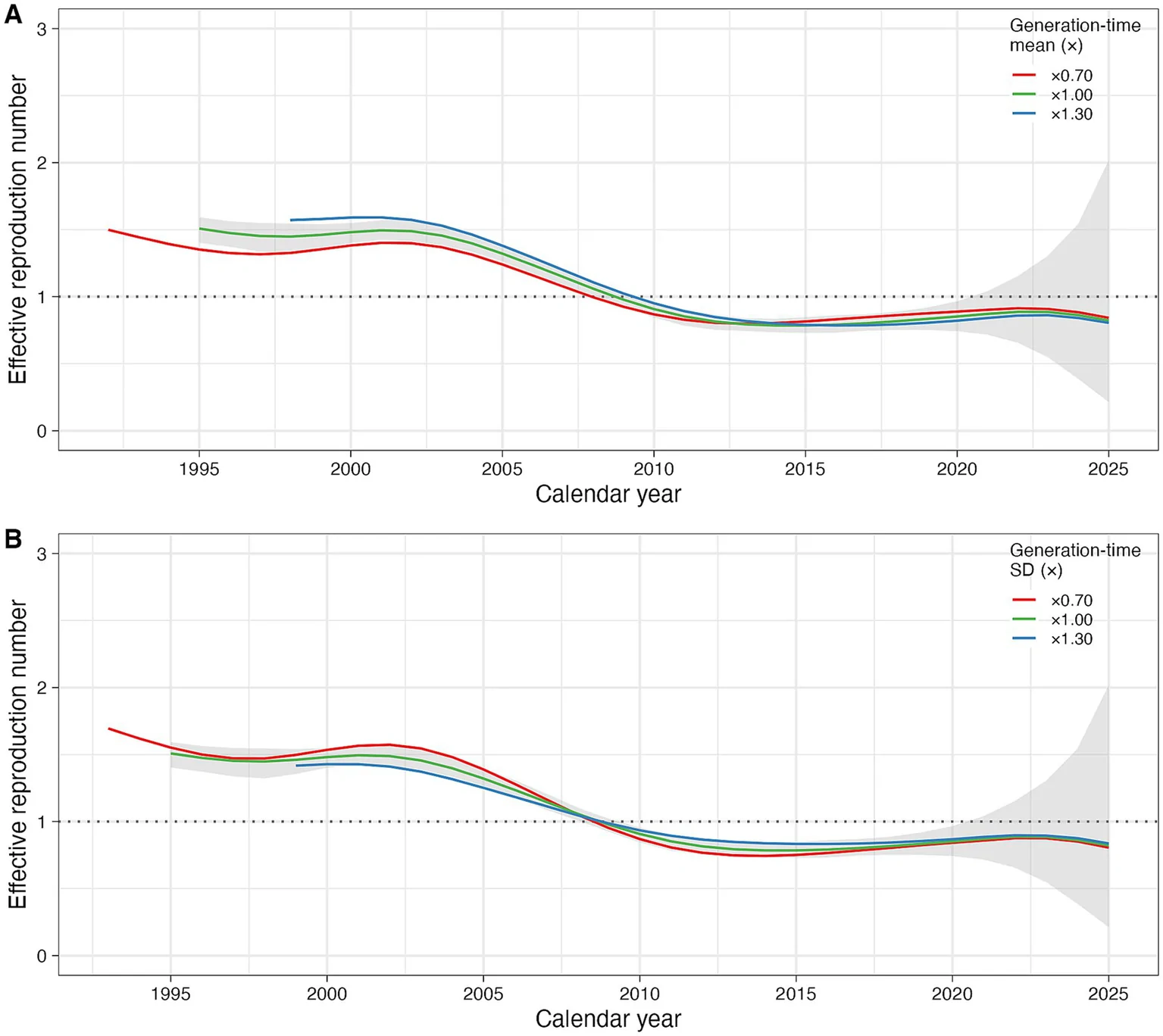
Sensitivity of *R*(*t*) to generation time. **(A)** Variation of the mean generation time by factors of 0.70–1.30. **(B)** Variations of the standard deviation of the generation time by the same factors as in Panel **(A)**, while holding the mean fixed. Colored lines are posterior medians for each scenario, and the gray band is the 95% credible interval of the baseline. The dotted horizontal line marks *R*(*t*) = 1.

## Discussion

The present study estimated the effective reproduction number *R*(*t*) of HIV infections among MSM, confirming that an increasing trend [with *R*(*t*) > 1] decreased to *R*(*t*) < 1 around 2008–2010. Using data through 2025, we found that the median *R*(*t*) has remained consistently below 1 since 2009, including during the COVID-19 pandemic. The latest *R*(*t*) estimate was the most uncertain, which was similar to our incidence estimation using the back-calculation technique. *R*(*t*) < 1 was found in major metropolitan areas, including Tokyo, Kanto-Koshinetsu, and Kinki, where the 95% CrI of *R*(*t*)—including the upper bound—fell below 1 during the declining phase of the epidemic. In addition, diagnostic performance in these metropolitan areas has been reported to be better than in other regions of Japan (33).

By estimating *R*(*t*) over time in Japan, our results suggest that control of HIV/AIDS has been successful overall. Even when the COVID-19 pandemic period was included, the long-term trend of *R*(*t*) remained unaffected. However, this was not the case for the diagnostic effort *α*(*t*), which showed a decline starting from shortly before the pandemic, and during the pandemic it is known that local health centers were overwhelmed by excessive number of tasks associated with COVID-19 (8). In addition, the estimated proportion of diagnosed HIV-infected individuals increased only slowly in recent years and failed to reach 95% or even 90%. Therefore, the steady trend of *R*(*t*) < 1, along with monotonically decreasing incidence in recent years (including during the COVID-19 pandemic), may be attributed to reasons other than testing and diagnosis, such as the recent increase in the coverage of pre-exposure prophylaxis.

The effective reproduction number has rarely been estimated for HIV, and even among the few settings where *R*(*t*) is available, our observation of a maintained *R*(*t*) < 1 for an extended period is consistent with published notions (27). Among MSM in Denmark and Sweden, the actual reproduction number peaked at the value of about 15 in 1981–1982 and then fell below 1 from 1988 to 1995 (11). In contrast, the *R*(*t*) of MSM transmission clusters in the Netherlands stayed at 1 over a long period, with 75% of clusters exceeding 1 between 2006 and 2010, indicating persistent and high transmission (34). Compared with these values, Japan’s HIV epidemic in MSM, which showed *R*(*t*) crossing the value of 1 around 2008–2010 and its median value subsequently remaining below 1, imply successful multisector support toward the high-risk group and continuously improving risk communication.

While our *R*(*t*) was practically useful, the back-calculation was structurally uncertain when inferring the dynamics for the latest calendar time, as recently infected individuals may not yet be substantially diagnosed. Thus, the most recent incidence of HIV infection can only be explored as later diagnosed cases are substantially observed, hence, the latest years exhibit the greatest uncertainty (13, 35). While a penalized-spline formulation can mitigate this terminal instability, it cannot always eliminate it (36, 37). Therefore, we also considered the piecewise constant model as an alternative approach, as it produced narrower credible intervals. This model assumes that *λ*(*t*) and *α*(*t*) remains to take a constant value within each calendar-time block, so that the block structure is part of the model specification rather than a quantity estimated from the data. The approximate constant structure within each interval might be regarded as a disadvantage that does not adhere to reality, and the piecewise constant model also suffers from substantial uncertainty in the latest incidence estimate, but our comparison indicated that the piecewise constant model outperformed the spline. As a possible countermeasure to remaining uncertainties, we have shown that the incidence-to-prevalence ratio can be jointly estimated alongside *R*(*t*) as an additional indicator of recent trends. To regularly monitor the transmission dynamics of HIV/AIDS, the most recent *R*(*t*) should be interpreted with caution (i.e., always consider the uncertainty) and be read as preliminary finding. For the sake of monitoring, sequential updates of estimates should be carried out.

While we explored a hierarchical model that accounted for a spatial dependent structure, the model that independently analyzed each region was shown to have performed the best. This result is not surprising, because a published phylogenetic study in Japan has indicated that the HIV-transmission networks among MSM are relatively closed within each geographic region and with limited intercity spread (38). However, the absence of geographic dependence in our model comparison does not necessarily demonstrate that regional epidemic in Japan is truly independent from each other. This is because spatial coupling between regions operates through human mobility and sexual networks rather than geographic adjacency. Therefore, it is possible that the mechanistic details of spatial transmission need to be understood using additional pieces of data. Furthermore, the pattern of geographic dependence has been shown to differ by country; e.g., in France, a large cluster centered in Paris spread to other regions (39), while in the Netherlands the epidemic was nationally dispersed (34), and in Poland networks linking cities were identified (40). Such spread is not indicative of uniform mixing but, rather, episodic bursts via the detailed local contact network (41). Role versatility (42) makes mixing within these local networks efficient, while nationwide, app-based partner-seeking (43) provides a sparse channel for hub-mediated coupling.

There were three technical limitations of this study. First, a considerable uncertainty in the most recent years was an intrinsic feature of back-calculation in which recent infections were only weakly determined until the time corresponding diagnoses of infection are substantially observed (35). While penalized-spline formulations can mitigate a portion of this uncertainty, they cannot completely eliminate this end-of-series effect (36). Hence, sequential, annual re-estimations are vital to fully interpret the most recent *R*(*t*) values (36). Second, this study was restricted to men in Japan. We excluded women and non-Japanese nationals, and the HIV epidemic was assumed to be largely closed and restricted to men-to-men contact. For this reason, our analytical framework cannot be applied to other populations in which our assumptions do not apply. Third, our approach still missed opportunities to fully quantify the uncertainty. For instance, despite the use of Bayesian method, we ignored the parametric uncertainties surrounding the generation time and incubation period. Another important task in the future may be to consider a stochastic analogue of the proposed method, allowing us to capture demographic stochasticity with small number of observed infections and exploring the transmission around the time that we finally aim to eliminate HIV/AIDS.

However, despite these limitations, we believe that the epidemic trends and regional variations were successfully captured from the existing surveillance data. In this study, the *R*(*t*) of HIV infection among MSM in Japan was estimated, demonstrating a continuous *R*(*t*) value <1 starting from approximately 2008–2010. Our *R*(*t*) estimates suggest that control of HIV/AIDS in Japan has been successful over the long term, including during the COVID-19 pandemic. However, the most recent estimates of *R*(*t*) were shown to be highly uncertain, highlighting the need for regular updates. In addition, the diagnostic rate *α*(*t*) declined; a trend that started shortly before the COVID-19 pandemic. As a consequence, the estimated proportion of HIV-infected individuals who have been diagnosed has increased only slowly in recent years, not at all reaching 95% and even not satisfying 90% at the end of 2025. Therefore, improving the testing and treatment strategies in Japan is critical.

## Data Availability

The datasets analyzed in this study are publicly available. The national HIV/AIDS surveillance data are published by the Committee of AIDS Trends, Ministry of Health, Labour and Welfare, Japan (https://api-net.jfap.or.jp/status/japan/nenpo.html). The code is available as Supplementary material.
