# Supplementary material for: Estimating the effective reproduction number of HIV infections in Japan from 1985 to 2025: a Bayesian extended back calculation
Source: Front Public Health. 2026 Oct 27;14:1944492. doi: 10.3389/fpubh.2026.1944492 (PMC13748209; doi:10.3389/fpubh.2026.1944492)
Supplement: Supplementary file 1 [file Data_Sheet_2.docx]

---

title: "Bayesian HIV back-calculation (spline alpha(t) version, main analysis)"

author: "Seiko Fujiwara"

date: "2026-06-23"

output:

html_document:

toc: true

toc_float: true

number_sections: true

df_print: default

---

<!-- File: HIV_backcalc_national_driver.Rmd -->

```{r setup, include=FALSE}

knitr::opts_chunk$set(echo = TRUE, message = FALSE, warning = FALSE, dpi = 130)

```

Both lambda(t) (new infections) and alpha(t) (diagnosis rate) are time-varying, each with a RW2 P-spline penalty

(Eilers & Marx 1996 / Lang & Brezger 2004; prior on sigma from Gelman 2006).

The model itself lives in the external file `HIV_backcalc_national_model.stan`; this driver compiles and runs it.

\# Packages, paths

```{r packages}

suppressMessages({

library(splines)	# B-spline basis bs()

library(cmdstanr)	# Stan

library(posterior)	# extract posterior draws

})

here	<- "."	# this package folder (set the working directory here before running)

code_dir <- here	# outputs (CSV) are written to this folder

```

\# Dataset

Japanese nationality, male, all transmission routes (main analysis; accounts for under-reporting of same-sex transmission).

```{r dataset}

data_csv <- file.path(here, "HIV_AIDS_JPmale_data_1985-2025.csv")

cat(sprintf("data: %s\n", data_csv))

```

\# Data, latency, basis, generation-time distribution

Read the observations (HIV diagnoses, AIDS onsets),

build survival S_rho and onset pmf f_rho from the Weibull AIDS latency, and assemble the shared B-spline basis

for lambda and alpha plus the fixed part of the generation-time distribution.

```{r data-latency-basis-kernel}

\# (1a) read data

dat <- read.csv(data_csv, fileEncoding = "UTF-8-BOM")

years	<- dat$Time	# calendar year 1985-2025

hiv_obs <- dat$HIV	# HIV new diagnoses that year (observed)

aids_obs <- dat$AIDS	# AIDS new onsets that year (observed)

n_year	<- nrow(dat)	# number of observed years (= 41)

\# (1b) Weibull AIDS latency (infection -> onset) -> survival S_rho and onset pmf f_rho

eta_weibull <- 11.5958011	# Weibull scale

k_weibull	<- 2.516	# Weibull shape

S_aidsfree <- numeric(n_year + 1) # S[kk] = still AIDS-free s=(kk-1) years after infection

for (kk in 1:(n_year + 1)) {

s <- kk - 1

S_aidsfree[kk] <- exp(-(s / eta_weibull)^k_weibull)

}

surv_aidsfree <- numeric(n_year)	# S_rho(s): AIDS-free survival up to s years

pmf_aids	<- numeric(n_year)	# f_rho(s): onset exactly at s years (difference of S_rho)

for (s in 1:n_year) {

surv_aidsfree[s] <- S_aidsfree[s + 1]

pmf_aids[s]	<- S_aidsfree[s] - S_aidsfree[s + 1]

}

\# (1c) shared cubic B-spline basis for lambda and alpha (df=12, 8 interior knots)

n_basis <- 12

B <- bs(1:n_year, df = n_basis, degree = 3, intercept = TRUE)

B <- matrix(as.numeric(B), n_year, n_basis)

\# (1d) fixed part of the generation-time distribution kernel_base (yearly integral of beta*S_rho; alpha is applied on the Stan side)

abu_beta	<- c(0.0107, 0.0008, 0.0042) * c(10.6, 11.0, 7.1) * 12	# infectiousness beta(s) by infection age

phase_breaks <- c(0, 2.5/12, 2.5/12 + 7.59, 2.5/12 + 7.59 + 2.0) # acute/chronic/late boundaries (years)

s_grid	<- seq(0.01, 12, by = 1/52)	# time since infection s, weekly grid

beta_grid <- ifelse(s_grid < phase_breaks[2], abu_beta[1],

ifelse(s_grid < phase_breaks[3], abu_beta[2],

ifelse(s_grid < phase_breaks[4], abu_beta[3], 0)))

S_aidsfree_grid <- exp(-(s_grid / eta_weibull)^k_weibull)

K_g <- 10	# truncation length of the generation time

\#	beta(s) is zero for infection ages beyond 9.798 years, so the weights vanish for s >= 11.

\#	K_g = 10 is therefore the smallest value that discards no probability mass, and it makes

\#	R(t) identifiable two years earlier (from 1995 instead of 1997).

kernel_base <- sapply(1:K_g, function(kk) { # integrate beta*S_rho over each 1-year bin k

in_bin <- s_grid >= (kk - 1) & s_grid < kk

sum(beta_grid[in_bin] * S_aidsfree_grid[in_bin])

})

\# (1e) data list passed to Stan

n_burn <- 0	# 0 = include all years in the likelihood

stan_data <- list(n_year = n_year, n_basis = n_basis, K_g = K_g, n_burn = n_burn,

hiv_obs = hiv_obs, aids_obs = aids_obs, B = B,

surv_aidsfree = surv_aidsfree, pmf_aids = pmf_aids, kernel_base = kernel_base)

```

\# Initial values

Inits only set where MCMC starts; if the model is robust they don't move the posterior.

So flat constants everywhere; only the order of magnitude has to match:

log lambda ~ 6 (lambda a few hundred) -> start theta at 6.

- theta (coefficients of log lambda): all 6 (log lambda ~ 6, lambda ~ 400, flat)

- theta_alpha (coefficients of alpha): all logit(0.1) (alpha = 0.1, flat)

- sigma: 0.5

```{r init}

\# set all initial values to flat constants (does not affect the answer; only the order of magnitude matches the data)

init_fun <- function() list(theta = rep(6, n_basis), # log lambda ~ 6 (lambda ~ 400), flat

theta_alpha = rep(qlogis(0.10), n_basis), # alpha = 0.1 (logit(0.1)), flat

sigma_theta = 0.5, sigma_alpha = 0.5)	# sigma = 0.5

```

\# Compile & sample

Compile the external .stan and sample with NUTS (the automatic HMC).

adapt_delta is raised from the default 0.8 to 0.95 to suppress divergences.

- $sample() usage ... cmdstanr docs

- NUTS ... Hoffman & Gelman 2014 (intuition: Betancourt 2017)

- adapt_delta / max_treedepth ... Stan warning guide

> Vehtari 2021 for R-hat/ESS theory <https://doi.org/10.1214/20-BA1221>

```{r sample}

mod <- cmdstan_model(file.path(here, "HIV_backcalc_national_model.stan"))

\# ★ MCMC samples from the joint posterior (defined in the model block of the .stan).

\#	each draw (1 row) of fit = one point over all 26 params at once → the theta correlations are inside it too.

fit <- mod$sample(data = stan_data, init = init_fun, chains = 4, parallel_chains = 4,

iter_warmup = 1000, iter_sampling = 1000, adapt_delta = 0.95, max_treedepth = 12,

seed = 2026, refresh = 250)

```

\# Convergence diagnostics (printed to console)

rhat (< 1.01 for convergence), ess_bulk/ess_tail (effective sample size). Also check the number of divergences and max_treedepth hits.

- rhat / ess ... Vehtari et al. 2021 (implemented in posterior)

- num_divergent / treedepth ... Stan warning guide

```{r convergence-console}

cat("\n=== convergence diagnostics (sigma, representative coefficients) ===\n")

print(as.data.frame(fit$summary(c("sigma_theta", "sigma_alpha", "theta[1]", "theta[12]", "theta_alpha[1]", "theta_alpha[12]"),

"mean", "sd", "rhat", "ess_bulk"), n = 20))

dg <- fit$diagnostic_summary()

cat("\nnum_divergent:", sum(dg$num_divergent), " | max_treedepth_hit:", sum(dg$num_max_treedepth), "\n")

```

\# Posterior summary

Build credible intervals (CrI) from the posterior quantiles. `band()` returns each yearly quantity as year x 5 columns (lo95/lo50/med/hi50/hi95).

```{r posterior-summary}

band <- function(varname) {

s <- fit$summary(sprintf("%s[%d]", varname, 1:n_year), ~quantile(.x, c(.025, .25, .5, .75, .975)))

q <- as.data.frame(s[, 2:6])

names(q) <- c("lo95", "lo50", "med", "hi50", "hi95")

cbind(year = years, q)

}

lamB <- band("lambda")	# band of lambda(t)

alpB <- band("alpha")	# band of alpha(t)

RB	<- band("R")	# band of R(t)

show <- c(1990, 2000, 2010, 2015, 2020, 2024)

idx <- match(show, years)

cat("\n=== alpha(t), R(t) posterior median [95% CrI] ===\n")

for (j in idx) {

cat(sprintf("%d alpha=%.3f [%.3f, %.3f] R=%s\n", years[j], alpB$med[j], alpB$lo95[j], alpB$hi95[j],

if (RB$med[j] == 0) " - " else sprintf("%.2f [%.2f, %.2f]", RB$med[j], RB$lo95[j], RB$hi95[j])))

}

```

\# Identifiability check: posterior correlation of lambda(t) and alpha(t)

A strong negative posterior correlation between lambda and alpha = a "trade-off" = possible weak identifiability.

```{r identifiability-corr}

draws <- as_draws_df(fit$draws()) # ★ each row = one sample from the joint posterior (all params)

corr_year <- sapply(seq_len(n_year), function(t) {

lv <- draws[[sprintf("lambda[%d]", t)]]

av <- draws[[sprintf("alpha[%d]", t)]]

cor(lv, av)

})

cat("\n=== lambda(t)-alpha(t) posterior correlation (strong negative = trade-off = sign of weak identifiability) ===\n")

for (j in idx) cat(sprintf("%d cor(lambda,alpha)=% .2f\n", years[j], corr_year[j]))

```

\# Estimates CSV

Save the yearly estimate table (median + 95% CrI of lambda, alpha, R, plus the lambda-alpha correlation) to one CSV.

```{r save-csv}

out <- data.frame(year = years,

alpha_med = alpB$med, alpha_lo = alpB$lo95, alpha_hi = alpB$hi95,

lambda_med = lamB$med, lambda_lo = lamB$lo95, lambda_hi = lamB$hi95,

R_med = RB$med, R_lo = RB$lo95, R_hi = RB$hi95,

cor_lambda_alpha = corr_year)

write.csv(out, file.path(code_dir, "HIV_backcalc_national_estimates.csv"), row.names = FALSE)

cat("\nsaved: HIV_backcalc_national_estimates.csv\n")

out

```

// File: HIV_backcalc_national_model.stan

// Bayesian HIV back-calculation: both lambda(t) and alpha(t) as cubic B-splines.

// R(t) is derived in generated quantities as a renewal-equation ratio from lambda and alpha(t), per posterior draw.

// Penalty = RW2 (second-order difference) smoothing prior = Bayesian P-spline:

//	Eilers & Marx 1996 Stat Sci 11(2):89-121	https://doi.org/10.1214/ss/1038425655

//	Lang & Brezger 2004 JCGS 13:183-212	https://doi.org/10.1198/1061860043010

//	half-Cauchy on sigma: Gelman 2006 Bayesian Anal 1(3):515-534 https://doi.org/10.1214/06-BA117A

data {

int<lower=1> n_year;

int<lower=1> n_basis;

int<lower=1> K_g;

int<lower=0, upper=n_year> n_burn;

array[n_year] int<lower=0> hiv_obs;

array[n_year] int<lower=0> aids_obs;

matrix[n_year, n_basis] B; // shared cubic B-spline basis for lambda and alpha

vector[n_year] surv_aidsfree; // S_rho(s)

vector[n_year] pmf_aids; // f_rho(s)

vector[K_g] kernel_base; // yearly integral of beta(k)*S_rho(k) (alpha-independent fixed part)

}

parameters {

vector[n_basis] theta; // coefficients of log lambda(t)

vector[n_basis] theta_alpha; // coefficients of logit alpha(t)

real<lower=0> sigma_theta; // RW2 smoothing SD for lambda

real<lower=0> sigma_alpha; // RW2 smoothing SD for alpha

}

transformed parameters {

vector[n_year] lambda = exp(B * theta);

vector[n_year] alpha = inv_logit(B * theta_alpha); // alpha(t) in (0,1) for each year

vector[n_year] E_HIV = rep_vector(0, n_year);

vector[n_year] E_AIDS = rep_vector(0, n_year);

// E_HIV: infections in year i-s that stay undiagnosed and AIDS-free for s-1 years, then diagnosed in year i at rate alpha(i)

for (i in 2:n_year) {

real surv = 1; // fraction still undiagnosed (accumulates as s grows)

for (s in 1:(i - 1)) {

E_HIV[i] += lambda[i - s] * surv * surv_aidsfree[s] * alpha[i];

surv *= (1 - alpha[i - s]); // apply the escape factor for the next s (one-year-older infection)

}

}

// E_AIDS: infections in year i-s that stay undiagnosed for s-1 years, then progress to AIDS in year i

for (i in 2:n_year) {

real surv = 1; // fraction still undiagnosed (accumulates as s grows)

for (s in 1:(i - 1)) {

E_AIDS[i] += lambda[i - s] * surv * pmf_aids[s];

surv *= (1 - alpha[i - s]); // apply the escape factor for the next s (one-year-older infection)

}

}

}

model {

// ★【the joint posterior is defined HERE】★

//	whole block (priors below × Poisson likelihood) = joint posterior of all params

//	(theta 12, theta_alpha 12, sigma_theta, sigma_alpha), up to a constant. no single line sets it.

//	NUTS samples this; each draw (1 row) in the driver = one point over all params at once.

//	→ lambda(t)=exp(B*theta) is per draw, so the lambda band carries the theta corr (not indep propagation).

// --- lambda(t): RW2 P-spline penalty ---

theta[1] ~ normal(0, 10);

theta[2] ~ normal(0, 10);

theta[3:n_basis] ~ normal(2 * theta[2:(n_basis - 1)] - theta[1:(n_basis - 2)], sigma_theta);

sigma_theta ~ cauchy(0, 1);

// --- alpha(t): same RW2 P-spline penalty as lambda (level held loosely near logit(0.1)) ---

theta_alpha[1] ~ normal(logit(0.1), 1); theta_alpha[2] ~ normal(logit(0.1), 1);

theta_alpha[3:n_basis] ~ normal(2 * theta_alpha[2:(n_basis - 1)] - theta_alpha[1:(n_basis - 2)], sigma_alpha);

sigma_alpha ~ cauchy(0, 1);

// --- Poisson likelihood ---

for (i in (n_burn + 1):n_year) {

target += poisson_lpmf(hiv_obs[i] | fmax(E_HIV[i], 1e-8));

target += poisson_lpmf(aids_obs[i] | fmax(E_AIDS[i], 1e-8));

}

}

generated quantities {

// The generation-time distribution g(t,k) is also evaluated under time-varying alpha

// (undiagnosed survival of the cohort infected at t-k) -> R(t)

vector[n_year] R = rep_vector(0, n_year);

for (t in (K_g + 1):n_year) {

vector[K_g] g_raw;

real surv = 1;

for (k in 1:K_g) {

g_raw[k] = kernel_base[k] * surv; // product_{u=t-k+1}^{t-1}(1-alpha(u))

surv *= (1 - alpha[t - k]);

}

vector[K_g] g = g_raw / sum(g_raw);

real denom = 0;

for (k in 1:K_g) denom += g[k] * lambda[t - k];

if (denom > 0) R[t] = lambda[t] / denom;

}

// posterior predictive (for PPC) + pointwise log-likelihood (for LOO/WAIC)

array[n_year] int hiv_rep;

array[n_year] int aids_rep;

vector[2 * n_year] log_lik; // [1..n_year]=HIV, [n_year+1..]=AIDS

for (i in 1:n_year) {

hiv_rep[i] = poisson_rng(fmax(E_HIV[i], 1e-8));

aids_rep[i] = poisson_rng(fmax(E_AIDS[i], 1e-8));

log_lik[i]	= poisson_lpmf(hiv_obs[i] | fmax(E_HIV[i], 1e-8));

log_lik[n_year + i] = poisson_lpmf(aids_obs[i] | fmax(E_AIDS[i], 1e-8));

}

}
