# Supplementary material for: Estimating the effective reproduction number of HIV infections in Japan from 1985 to 2025: a Bayesian extended back calculation
Source: Front Public Health. 2026 Oct 27;14:1944492. doi: 10.3389/fpubh.2026.1944492 (PMC13748209; doi:10.3389/fpubh.2026.1944492)
Supplement: Supplementary file 2 [file Supplementary_file_1.docx]

**Supplement to: Estimating the effective reproduction number of HIV infections in Japan, 1985 to 2025: a Bayesian extended back-calculation**

**Table** **of contents**

1. Supplementary Methods
2. Supplementary Figures S1–S4
3. Supplementary Tables S1–S4

**Supplementary Methods**

**S1. Bayesian estimations and Markov chain Monte Carlo settings**

Posterior distributions were derived from the Hamiltonian Monte Carlo with the No-U-Turn Sampler method, implemented in Stan software through the cmdstanr interface (CmdStan 2.39.0). We ran four independent chains, each with 1,000 warmup iterations followed by 1,000 sampling iterations, with the target average acceptance probability set to *adapt_delta* = 0.95 and a fixed, random seed (*seed* = 2026). Convergence was assessed following Vehtari et al. (S1): the potential scale reduction factor was *R̂* < 1.01 for all parameters, and both the bulk and tail effective sample sizes exceeded the target of 400. The convergence diagnostics (*R̂* and effective samples sizes) met their targets (Figure S1), and trace plots showed no separation, sticking, or trend across the four chains (Figure S2). Estimates from the pooled posterior draws are reported in the main text as posterior medians with 95% credible intervals.

The annual numbers of HIV diagnoses and AIDS cases were dealt with as conditionally independent Poisson count-data model with expected values given by equations (1) and (2) in the main text. The likelihood was constructed as the product of the Poisson probabilities for both observed series across all 41 years from 1985 through 2025. The national model included 26 parameters in total: 12 spline coefficients for *λ*(*t*), 12 spline coefficients for *α*(*t*), and two standard deviations governing the degree of smoothing.

$${HIV}_{t} \sim Poisson\left( E\left[ {HIV}_{t} \right] \right), {AIDS}_{t} \sim Poisson\left( E\left[ {AIDS}_{t} \right] \right)$$

(S1)

$$L\left( \theta\right) = \prod_{t = 1985}^{2025} Pois\left( {HIV}_{t} | E\left[ {HIV}_{t} \right] \right) Pois\left( {AIDS}_{t} | E\left[ {AIDS}_{t} \right] \right)$$

(S2)

**S2. Derivation of the discretized McKendrick equation**

Let *h_t_*_,_*_s_* represent the number of undiagnosed HIV infected individuals who, in calendar year *t*, were infected *s* years ago and remain alive and free from AIDS. Infected individuals leave *h* either by HIV diagnosis or AIDS onset, occurring with annual probabilities *α_t_* and *ρ_s_*, respectively. The discrete-time McKendrick equation describing the evolution of *h_t_*_,_*_s_* is:

$$h_{t,s}=h_{t-1,s-1}\left( 1 - \alpha_{t - 1} \right)\left( 1 - \rho_{s} \right) \left( s \geq2 \right),$$

$$h_{t,1}=\lambda_{t - 1}\left( 1 - \rho_{1} \right), h_{t,0}=\lambda_{t},$$

(S3)

$$h_{t,s}= \lambda_{t - s}\prod_{x = t - s + 1}^{t - 1} \left( 1 - \alpha_{x} \right)\prod_{y = 1}^{s} \left( 1 - \rho_{y} \right) = \lambda_{t - s}S_{\alpha}\left( t, s \right)S_{\rho}\left( s \right)$$

(S4)

A more complete derivation from McKendrick partial differential equations, together with its continuous-time formulation, is found in Nishiura (S2).

**S3. Survival functions, transmission weights and undiagnosed prevalence**

AIDS-free survival was assumed to depend only on infection age and was modeled using a discrete Weibull distribution with scale *η* = 11.596 years and shape *k* = 2.516 (Table S1). In contrast, undiagnosed survival varies with calendar year and infection age, because the annual probability of diagnosis changes over calendar time. Both survival functions enter equation (1) in the main text. In the renewal equation, the transmission weights are obtained by normalizing the product of relative infectiousness, AIDS-free survival, and undiagnosed survival.

$$S_{\rho}\left( s \right) = exp\left\{ -\left( \frac{s}{\eta} \right)^{k} \right\}, f_{\rho}\left( s \right) = S_{\rho}\left( s - 1 \right) - S_{\rho}\left( s \right)$$

(S5)

$$S_{\alpha}\left( t, s \right) = \prod_{x = t - s + 1}^{t - 1} \left( 1 - \alpha_{x} \right)$$

(S6)

$$w_{t,s}=\frac{\beta\left( s \right)S_{\rho}\left( s \right)S_{\alpha}\left( t, s \right)}{\sum_{u = 1}^{s_{max}} \beta\left( u \right)S_{\rho}\left( u \right)S_{\alpha}\left( t, u \right)}, s = 1, \ldots, s_{max}$$

(S7)

The prevalence of undiagnosed HIV infected individuals represents the number of individuals who have been infected with HIV but, by the end of year *t*, have neither developed AIDS nor been diagnosed as HIV infected.

$$X\left( t \right) = \lambda_{t} + \sum_{s = 1}^{t - 1} \lambda_{t - s}S_{\rho}\left( s \right)\prod_{x = t - s + 1}^{t} \left( 1 - \alpha_{x} \right)$$

(S8)

**S4. Spline representation and smoothing priors**

The annual number of new HIV infections *λ*(*t*) and the yearly diagnosis rate *α*(*t*) were modeled as smooth functions of calendar time using a cubic B-spline basis, *B,* with 12 basis functions:

$$\lambda(t) = exp(B\theta^{\lambda}), \alpha(t) = {logit}^{-1}(B\theta^{\alpha}).$$

(S9)

The spline coefficients *θ^λ^* and *θ^α^* were assigned second-order random-walk (RW2) smoothing priors, and the smoothing strengths (the RW2 innovation standard deviations) were estimated from the data, giving a Bayesian penalized-spline (P-spline) formulation (S3).

More specifically, separate second-order random-walk priors were assigned to the spline coefficients *θ_λ_* and *θ_α_*, with innovation standard deviations *σ_λ_* and *σ_α_*, respectively. The first two coefficients in each vector were given weakly informative normal priors, while half-Cauchy(0, 1) priors were assigned to both smoothing standard deviations, following Gelman (S4).

$$\theta_{j} \sim N\left( 2\theta_{j - 1} - \theta_{j - 2}, \sigma\right), j = 3, \ldots, 12$$

(S10)

**S5. Sensitivity analysis with respect to generation time**

We assessed robustness to the assumed generation-time distribution *g* = *βS_ρ_S_α_*. Relative to the baseline *βS_ρ_* (mean *m*_0_ ≈ 4.54 years, standard deviation *SD*_0_ ≈ 3.45 years), the mean was scaled by factors of 0.70, 1.00, and 1.30 and (independently) the standard deviation was scaled by the same factors. *R*(*t*) was estimated under each of the resulting scenarios.

**S6. Piecewise constant model for *λ*(*t*) and *α*(*t*)**

To quantify the impact of smoothing effect on the latest estimate of R(t), we replaced the B-spline model in equation (S9) with a piecewise constant model. Specifically, *λ*(*t*) and *α*(*t*) were assumed to remain constant within each calendar-time interval. The observation period was partitioned into four-year intervals from 1985 through 2016 and three-year intervals from 2017 onward.

**S7. Regional hierarchical model and spatial structure (scaled Besag-York-Mollié: BYM2)**

The national back-calculation was extended to eight regional blocks. For each block *r*, region-specific deviations were added to the spline linear predictors of the log incidence and the diagnosis rate, so that regional dynamics varied around the shared national mean. We compared three structures for the regional deviations *b_r_*: (i) a nonspatial model, in which *b_r_* were independent and identically distributed Gaussian random effects; and two spatial models for which neighboring regions were correlated using (ii) geographic adjacency and (iii) men who have sex with men (MSM)-venue adjacency. Both spatial models used the scaled Besag-York-Mollié specification, i.e., the BYM2 reparameterization of Riebler et al. (S5).

In the BYM2 parameterization, each deviation combined an unstructured (independent) component and a spatially structured component through the single mixing proportion *φ* ∈ [0, 1] and the marginal precision *τ*:

$$b_{r} = \frac{1}{\sqrt{\tau}}(\sqrt{(1 - \varphi) v_{r}} + \sqrt{\varphi{u^{*}}_{r}}),$$

(S11)

where *v_r_* ~ Normal (0, 1) was the unstructured effect and *u*^*^*_r_*, the intrinsic conditional autoregressive (ICAR) component defined on the regional adjacency graph and rescaled to have unit generalized variance. This scaling made the mixing proportion *φ* interpretable as the proportion of the marginal variance and was spatially structured comparably across adjacency graphs: *φ* ≈ 0 corresponded to a purely unstructured (nonspatial) model and *φ* ≈ 1 to a purely spatial one.

We assigned a Beta (0.5, 0.5) prior to the mixing proportion *φ*, a half-normal prior to the marginal scale, and imposed a soft sum-to-zero constraint on the ICAR component.

Two adjacency graphs were examined: geographic adjacency, which linked regional blocks that shared a land border, and MSM-venue adjacency, linking blocks connected through the nationwide distribution of MSM-venues. The three candidate models were fitted to the same 640 observation points (annual HIV and AIDS diagnoses for the eight regions from 1985 through 2024) and compared by the widely applicable information criterion (WAIC), the Bayesian information criterion (BIC), and leave-one-out cross-validation. In both spatial models, the posterior of the spatially structured share *φ* concentrated near zero, and the nonspatial model gave the most parsimonious fit and was adopted for the main analysis. The full comparison is reported in Table 1 in the main text.

S8. Comparison between the spline model and the piecewise constant model

For each cut-off year from 2021 to 2024, the estimate of *R*(*t*) for in the latest year was compared with the corresponding *R*(*t*) obtained using the observed data up to the end of 2025. The comparison was based on the three measures defined in Table S3, which assess the validity, reliability and performance of spline and piecewise constant models (Figure S4).

The length of calendar-time steps in the piecewise constant model was evaluated using leave-one-out cross-validation, the widely applicable information criterion (WAIC) and the Bayesian information criterion (BIC). Models with uniform steps of one, two, three and five years, together with the specification adopted in the main analysis (four-year intervals from 1985 through 2016 and three-year intervals from 2017 onward), were fitted to the observed data through 2025. For all three criteria, the log-likelihood was calculated from the annual numbers of HIV diagnoses and AIDS cases (Table S4).

Supplementary Tables

**Table S1. Fixed variables and parameters.**

| **Quantity** | **Infection age or parameter** | **Value** | **Source** |
| --- | --- | --- | --- |
| Relative infectiousness *β*(*s*), acute phase | 0 ≤ *s* < 0.208 years | 1.361 per year (0.0107 per act × 10.6 acts per month × 12) | (S6, S7) |
| Relative infectiousness *β*(*s*), chronic phase | 0.208 ≤ *s* < 7.798 years | 0.106 per year (0.0008 × 11.0 × 12) | (S6, S7) |
| Relative infectiousness *β*(*s*), late phase | 7.798 ≤ s < 9.798 years | 0.358 per year (0.0042 × 7.1 × 12) | (S6, S7) |
| Relative infectiousness *β*(*s*), beyond the late phase | *s* ≥ 9.798 years | 0 | (S6, S7) |
| Incubation period, Weibull scale *η* | – | 11.596 years | (S8, S9, S10) |
| Incubation period, Weibull shape *k* | – | 2.516 | (S8, S9, S10) |
| Implied median incubation period | *η* (ln 2)^(1/*k*) | 10.02 years | Derived |

Relative infectiousness *β*(*s*) was expressed on an annual scale and was calculated as the product of the per-act transmission probability, the number of acts per month, and the duration 12 months; the component values used in this calculation are shown in parentheses. The transmission kernel was truncated at an infection age of *s*_max_ years (the default *s*_max_ was set at 10 years and technically taking greater value of *s*_max_ did not change the results).

**Table S2. Functions and variables that were quantified and fixed.**

| **Quantity** | **Estimated or fixed** | **Specified in** |
| --- | --- | --- |
| *λ*(*t*), yearly incidence of new HIV infections | Estimated (penalized B-spline) | Equation (S9) |
| *α*(*t*), yearly probability of HIV diagnosis | Estimated (penalized B-spline) | Equation (S9) |
| *ρ_s_*, yearly hazard of AIDS onset | Fixed | Table S1 |
| *β(s)*, relative infectiousness | Fixed | Table S1 |
| *S_ρ_(s)*, AIDS-free survival | Derived | Section S3 |
| *S_α_(t,s)*, undiagnosed survival | Derived | Section S3 |
| *w_s,t_*, transmission weight | Derived | Section S3 |
| *X(t)*, undiagnosed prevalence | Derived | Section S3 |
| *R(t)*, effective reproduction number | Derived | Equation (3) |

**Table S3. Comparison of the effective reproduction number obtained in the past against that obtained using the dataset up to 2025.**

| **Model for *λ*(*t*) and *α*(*t*)** | **Root of squared errors** | **Coverage probability** | **Interval score** |
| --- | --- | --- | --- |
| Spline model (Figure 2A) | 0.522 | 4/4 (100%) | 2.860 |
| Piecewise constant model (Figure 2B) | 0.174 | 4/4 (100%) | 0.488 |

For each cut-off year *t* from 2021 to 2024, the estimate of *R*(*t*) in the latest year was compared with the estimate of *R*(*t*) of the same calendar year based on the data up to the end of 2025. The coverage probability shows the proportion of past estimation that contained the updated posterior median *R*(*t*) of the same year within the 95% credible intervals. The interval score was based on the same comparison, and was calculated as (*U* − *L*) + (2/*α*)(*L* − *y*)*I*(*y* < *L*) + (2/*α*)(*y* − *U*)*I*(*y* > *U*), with *α* = 0.05, where *L* and *U* denote the lower and upper bounds of the 95% CrI obtained in the past, and y is the posterior median using the data up to 2025. Smaller value indicates better performance.

**Table S4. Comparison of the length of the calendar-time blocks in the piecewise constant model.**

| **Model for *λ*(*t*) and *α*(*t*)** | **elpd_LOO** | **WAIC** | **BIC** | **Number of parameters** | **Interval score** |
| --- | --- | --- | --- | --- | --- |
| Piecewise constant, one-year blocks | -462.5 | 897.6 | 1174.7 | 82 | 9053 |
| Piecewise constant, two-year blocks | -506.0 | 968.8 | 1045.9 | 40 | 9220 |
| Piecewise constant, three-year blocks | -549.8 | 1072.6 | 1070.5 | 26 | 2427 |
| Piecewise constant, four- and three-year blocks (Figure 2B) | -523.6 | 1034.2 | 1048.4 | 22 | 0.488 |
| Piecewise constant, five-year blocks | -560.9 | 1116.6 | 1096.8 | 16 | 0.348 |

All models were fitted to the same set of observations through 2025. The interval score is defined in Table S3. For one-year blocks, the terminal block contains only a single year at every cut-off, making *R*(*t*) unidentifiable.

References

S1. Vehtari A, Gelman A, Simpson D, Carpenter B, Bürkner P-C. Rank-normalization, folding, and localization: an improved R̂ for assessing convergence of MCMC. Bayesian Anal (2021) 16:667–718. doi: 10.1214/20-BA1221

S2. Nishiura H. Estimating the incidence and diagnosed proportion of HIV infections in Japan: a statistical modeling study. PeerJ (2019) 7:e6275. doi: 10.7717/peerj.6275

S3. Hogan DR, Salomon JA. Spline-based modelling of trends in the force of HIV infection, with application to the UNAIDS Estimation and Projection Package. Sex Transm Infect (2012) 88(Suppl 2):i52–i57. doi: 10.1136/sextrans-2012-050652

S4. Gelman A. Prior distributions for variance parameters in hierarchical models. Bayesian Anal (2006) 1:515–534. doi: 10.1214/06-BA117A

S5. Riebler A, Sørbye SH, Simpson D, Rue H. An intuitive Bayesian spatial model for disease mapping that accounts for scaling. Stat Methods Med Res (2016) 25:1145–1165. doi: 10.1177/0962280216660421

S6. Abu-Raddad LJ, Longini IM. No HIV stage is dominant in driving the HIV epidemic in sub-Saharan Africa. AIDS (2008) 22:1055–1061. doi: 10.1097/QAD.0b013e3282f8af84

S7. Hollingsworth TD, Anderson RM, Fraser C. HIV-1 transmission, by stage of infection. J Infect Dis (2008) 198:687–693. doi: 10.1086/590501

S8. Brookmeyer R, Goedert JJ. Censoring in an epidemic with an application to hemophilia-associated AIDS. Biometrics (1989) 45:325–335. doi: 10.2307/2532057

S9. Boldsen JL, Jensen JL, Søgaard J, Sørensen M. On the incubation time distribution and the Danish AIDS data. J R Stat Soc Ser A (1988) 151:42–43. doi: 10.2307/2982183

S10. Muñoz A, Xu J. Models for the incubation of AIDS and variations according to age and period. Stat Med (1996) 15:2459–2473. doi: 10.1002/(SICI)1097-0258(19961130)15:22<2459::AID-SIM464>3.0.CO;2-Q

**Supplementary Figures**

**Figure S1. Convergence diagnostics for 26 model parameters (spline coefficients *θ_λ_* and *θ_α_*, smoothing SDs *σ_λ_* and *σ*_α_) from four chains.**

**(A)** $\hat{R}$, comparing the between- and within-chain variance (dashed line, 1.01). **(B)** Bulk and tail effective sample size (ESS; dashed line, 400). Bulk-ESS applies to the posterior mean and median, with tail-ESS to the 2.5% and 97.5% quantiles.

**Figure S2. Trace plots for all 26 parameters (spline coefficients *θ_λ_* and *θ_α_*, smoothing SDs *σ_λ_* and *σ*_α_) from four chains.**

New infections *λ*(t) and the diagnosis rate *α*(t) are each fitted as a penalized B-spline with 12 coefficients (*θ_λ_* and *θ_α_*). *σ_λ_* and *σ*_α_ are their smoothing standard deviations, i.e. the standard deviations of the second-order random-walk penalty on the coefficients (half-Cauchy (0, 1) prior). Each panel shows the four chains.

**Figure S3. Structurally weak identifiability between new infections *λ* and the diagnosis rate *α*.**

**(A)** The posterior correlation between *λ*(*t*) and *α*(*t*) by calendar year. **(B)** Joint posteriors of *λ* versus *α* for the years 2020 through 2025. Toward the present, the two quantities increasingly trade off, with the correlation falling below 0.7 and the joint posterior becoming elongated, reflecting the limited information available to separate them near the censoring boundary.

**Figure S4. Comparison of the effective reproduction number obtained in the past against the estimate using the dataset up to 2025.**

For each calendar year from 2021 to 2024, the red symbol shows the posterior median and 95% credible interval of *R*(*t*) obtained when the model was fitted to the series that ends in the corresponding calendar year, and the blue symbol shows the same estimate obtained when the model was fitted to the series that ends at the end of 2025. The left panel shows estimates from the spline model for *λ*(*t*) and *α*(*t*), and the right panel shows estimates from the piecewise constant model. The dotted horizontal line indicates *R*(*t*) = 1.
