# Supplementary material for: Estimating the effective reproduction number of HIV infections in Japan from 1985 to 2025: a Bayesian extended back calculation
Source: Front Public Health. 2026 Oct 27;14:1944492. doi: 10.3389/fpubh.2026.1944492 (PMC13748209; doi:10.3389/fpubh.2026.1944492)
Supplement: Supplementary file 3 [file Supplementary_file_2.DOCX]

**STROBE Statement—checklist of items that should be included in reports of observational studies**

Manuscript: Estimating the effective reproduction number of HIV infection in Japan, 1985 to 2025: a Bayesian extended back-calculation

*Page numbers refer to the corresponding manuscript sections; exact page numbers to be finalized on the typeset proof.*

| **Item No.** | **Recommendation** | **Page No.** |
| --- | --- | --- |
| **Title and abstract** | | |
| 1 | (a) Indicate the study's design with a commonly used term in the title or the abstract (b) Provide in the abstract an informative and balanced summary of what was done and what was found | Title; Abstract |
| **Introduction** | | |
| 2 | Background/rationale: Explain the scientific background and rationale for the investigation being reported | Introduction |
| 3 | Objectives: State specific objectives, including any prespecified hypotheses | Introduction (final paragraph) |
| **Methods** | | |
| 4 | Study design: Present key elements of study design early in the paper | Materials and Methods (opening) |
| 5 | Setting: Describe the setting, locations, and relevant dates, including periods of recruitment, exposure, follow-up, and data collection | Materials and Methods (Japan; 1985–2025; national notification system) |
| 6 | Participants: Give the eligibility criteria, and the sources and methods of selection of participants | Materials and Methods (Japanese nationals; men; HIV and AIDS notifications) |
| 7 | Variables: Clearly define all outcomes, exposures, predictors, potential confounders, and effect modifiers. Give diagnostic criteria, if applicable | Materials and Methods (new HIV infections λ(t), diagnosis rate α(t), R(t), diagnosed proportion; outcome = annual HIV/AIDS diagnosis counts) |
| 8 | Data sources/measurement: For each variable of interest, give sources of data and details of methods of assessment (measurement). Describe comparability of assessment methods if there is more than one group | Materials and Methods (national HIV/AIDS surveillance under the Infectious Disease Control Law) |
| 9 | Bias: Describe any efforts to address potential sources of bias | Materials and Methods; Discussion (under-reporting of same-sex transmission; terminal right-censoring) |
| 10 | Study size: Explain how the study size was arrived at | Materials and Methods (all notified cases, 1985–2025; complete enumeration, no sampling) |
| 11 | Quantitative variables: Explain how quantitative variables were handled in the analyses. If applicable, describe which groupings were chosen and why | Materials and Methods (λ(t) and α(t) as penalized B-splines of calendar time) |
| 12 | Statistical methods: (a) Describe all statistical methods, including those used to control for confounding; (b) Describe any methods used to examine subgroups and interactions; (c) Explain how missing data were addressed; (d) If applicable, describe analytical methods taking account of sampling strategy; (e) Describe any sensitivity analyses | Materials and Methods (Bayesian back-calculation; renewal equation; HMC/NUTS in Stan; regional hierarchical model; model comparison by LOO/WAIC). (c) No missing data (complete national annual counts). (d) N/A — complete enumeration, not a sample. (e) Generation-time sensitivity analysis |
| **Results** | | |
| 13 | Participants: (a) Report numbers of individuals at each stage of study; (b) Give reasons for non-participation at each stage; (c) Consider use of a flow diagram | N/A — aggregate annual notification counts, not individual-level data (25,815 HIV and 11,446 AIDS cases notified, 1985–2025). Stage-wise flow / flow diagram not applicable |
| 14 | Descriptive data: (a) Give characteristics of study participants and information on exposures and potential confounders; (b) Indicate number of participants with missing data for each variable of interest | Materials and Methods; Results (annual HIV and AIDS counts by year; no missing data) |
| 15 | Outcome data: Report numbers of outcome events or summary measures | Results (observed annual HIV and AIDS counts; posterior predictive check, Figure 1) |
| 16 | Main results: (a) Give unadjusted estimates and, if applicable, confounder-adjusted estimates and their precision (eg, 95% confidence interval); (b) Report category boundaries when continuous variables were categorized; (c) If relevant, consider translating estimates of relative risk into absolute risk | Results (R(t) posterior median with 95% CrI, national and regional; diagnosed proportion vs the 95% target) |
| 17 | Other analyses: Report other analyses done—eg analyses of subgroups and interactions, and sensitivity analyses | Results (spatial model comparison; generation-time sensitivity analysis) |
| **Discussion** | | |
| 18 | Key results: Summarise key results with reference to study objectives | Discussion (opening paragraph) |
| 19 | Limitations: Discuss limitations of the study, taking into account sources of potential bias or imprecision. Discuss both direction and magnitude of any potential bias | Discussion (terminal instability of back-calculation; the two stated limitations) |
| 20 | Interpretation: Give a cautious overall interpretation of results considering objectives, limitations, multiplicity of analyses, results from similar studies, and other relevant evidence | Discussion |
| 21 | Generalisability: Discuss the generalisability (external validity) of the study results | Discussion (restricted to men in Japan; between-region coupling through metropolitan hubs) |
| **Other information** | | |
| 22 | Funding: Give the source of funding and the role of the funders for the present study and, if applicable, for the original study on which the present article is based | Funding |

*An Explanation and Elaboration article discusses each checklist item and gives methodological background and published examples of transparent reporting. The STROBE checklist is best used in conjunction with this article (freely available at http://www.strobe-statement.org).*
